# Assessing Adequacy of Marker Ball Placement in Preoperative Pelvic X-rays: Are We Missing the Mark?

**DOI:** 10.7759/cureus.94736

**Published:** 2025-10-16

**Authors:** Adam Khan Rahim, Hassan Imtiaz, Izza Afzal, Arsallan Karim, Parth Shah, Kunjan Barot, Abdullah Durrani, Georgios Kouklidis, Talha Ahmed, Antony Raymond

**Affiliations:** 1 Trauma and Orthopaedics, University Hospitals Dorset NHS Foundation Trust, Poole, GBR; 2 ENT, University Hospitals Dorset NHS Foundation Trust, Poole, GBR

**Keywords:** hemiarthroplasty / total hip replacement, hip fracture, marker ball, preoperative templating, radiographic calibration

## Abstract

Introduction

Accurate preoperative templating in hip arthroplasty depends on correct marker ball placement during anteroposterior (AP) pelvic radiographs. Misplacement can compromise prediction of femoral offset and leg length, increasing operative complexity and complication risk. This closed-loop audit evaluated marker ball adequacy in patients with intracapsular neck of femur fractures and assessed the impact of targeted interventions.

Methods

A retrospective audit was conducted at Poole General Hospital, United Kingdom. Patients with radiologically confirmed intracapsular fractures between January and May 2025 were included; exclusions were extracapsular fractures, age <18, open fractures, and prior contralateral hip replacement. Audit standards required marker balls to be entirely visible and positioned between femurs, inferior to the symphysis pubis, with an agreed compliance target of 80%. Interventions included educational presentations, poster reminders, and a mandatory checklist. A re-audit was performed from June 1-30, 2025. Statistical analysis was performed using chi-square testing.

Results

Cycle one included 60 cases, with marker balls present in 45% (n=27). Of these, 59% (n=16) were entirely visible and 41% (n=11) were correctly positioned, yielding overall compliance of 59%. Following interventions, cycle two (n=50) demonstrated significant improvement: marker balls present in 78% (n=39), with 84% (n=33) entirely visible (p=0.042) and 82% (n=32) correctly positioned (p=0.0014). Overall compliance increased to 80%, meeting the target standard.

Conclusion

Simple, low-cost educational measures significantly improved marker ball placement in pelvic radiographs for hip fracture patients. Embedding such interventions into routine radiography practice enhances the accuracy of preoperative templating, promotes patient safety, and reduces medico-legal risk. Ongoing audit and education are essential to sustain improvements.

## Introduction

A hip fracture is defined as a break in the upper part of the femur (thigh bone), usually occurring at or near the femoral neck, the intertrochanteric region, or the subtrochanteric region. Based on the anatomical location, they are further divided into extracapsular and intracapsular fractures. Extracapsular fractures occur outside the capsule, extending from the intertrochanteric region to 5 cm below the lesser trochanter. Intracapsular fractures occur inside the capsule, between the edge of the femoral head and the insertion of the capsule at the intertrochanteric line, and are more troublesome due to the higher risk of non-union and risk of avascular necrosis of the head of femur [1]. 

In the United Kingdom, the proportion between extracapsular and intracapsular fractures is ~41% and ~59% respectively [2]. Broadly focusing on intracapsular neck of femur fractures, the choice of surgery is narrowed down to half hip replacement (hemi arthroplasty) vs total hip replacement (THR), however, intracapsular neck of femur fractures can also be managed with screws/two-hole dynamic hip screws (mostly in cases of young neck of femur/undisplaced fractures). The percentage prevalence between these former two options comes down to 50% and 25% respectively [3]. 

The basis of both these surgeries, namely hemi-arthroplasty and THR, is how accurately templating is done preoperatively by the surgeon for the respective stems that are to be used as an implant. There are broadly two stems used, namely the older collarless polished tapered (CPT; Zimmer Biomet, Warsaw, IN, USA) [4], and the newer Exeter stems (Stryker Orthopaedics, Mahwah, NJ, USA) [5]. 

**Figure 1:**
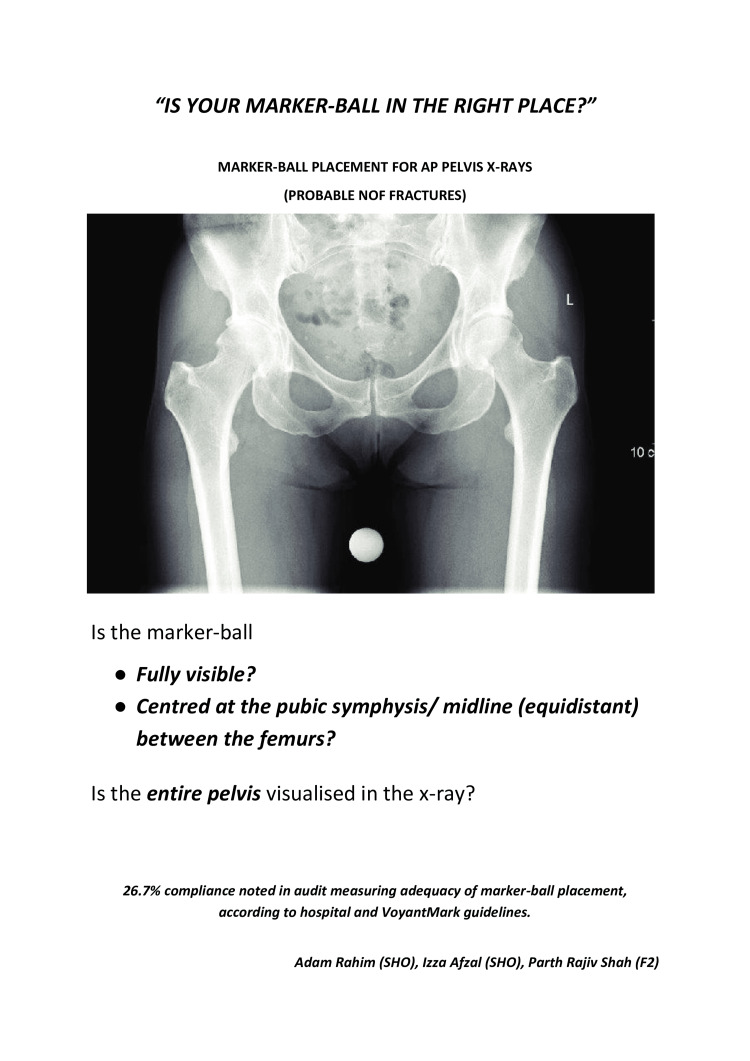
Appendix 1 (Marker ball poster for audit) Poster displayed in relevant clincial areas to increase awareness amongst radiographers to ensure appropriate marker ball placement while taking an anteroposterior view of the pelvis.

Templating for surgery is done by placing a marker ball in the adequate position, which is the presence of a marker ball between the femurs, inferior to the plane of symphysis pubis (Figure 1), when patients with suspected neck of femur fractures are undergoing diagnostic X-ray (anteroposterior (AP) view). The importance of the marker ball is that it is used to assess the offset of the implant stem which is to be used in the surgery. The ‘offset’ is defined as the horizontal distance from the centre of the femoral head (hip joint rotation centre) to a line drawn down the axis of the femoral shaft. Measuring the offset preoperatively allows better stability of the hip implant intraoperatively. It also helps in predicting placement of the stem in the bone to reduce the chance of leg length discrepancy [6]. Without accurate marker ball placement and templating, the offset would be difficult to predict and hence intraoperatively multiple trials would need to be attempted, which increases the risk of intraoperative fractures and prolonged anaesthetic time [7-9]. With the introduction of newer Exeter stems, marker ball use has become even more important as the magnification factors used for templating are solely dependent on the correct placement of marker balls [5]. 

We conducted a closed-loop audit at our trauma unit at a district general hospital to evaluate the adequacy of marker ball placement in preoperative pelvic X-rays and to assess the impact of educational and procedural interventions on improving compliance with placement standards.

## Materials and methods

This was a closed-loop audit conducted at our Poole General Hospital, United Kingdom. Patients presenting to accidents and emergency department post-trauma with a clinically suspected intracapsular neck of femur fracture were included. Patients who had a radiologically confirmed extracapsular neck of femur fracture, under the age of 18, those with open fractures, and those with previous contralateral hip replacement and pelvic hardware (due to high inaccuracy rate for templating) were excluded. Patients were identified from Electronic Clinical and Management Information Software (eCaMIS), a software that manages patient administrative details.

The audit standards were based on local trauma unit guidelines, which states that for those patients that have a marker ball present in their X-ray pelvis (AP view), the entirety of the marker needs to be visible, with some portion of the stem of the ball showing (‘stem’ refers to refers to the flexible neck or a similar attachment that allows the spherical marker to be positioned), and the marker ball must be placed in between the femurs, in an inferior plane to the symphysis pubis of the pelvis. Following discussion with the hip surgeons, an acceptable compliance of 80% was agreed upon. The standards are presented in Table 1.

**Table 1:** Audit Standards

Standard	Target compliance (%)
Marker ball visible entirely	80
Marker ball placed between both femurs and inferior to symphysis pubis	80

Data was collected on patient demographics, confirmation of intracapsular neck of femur fractures, the presence of a marker ball in X-ray pelvis (AP view), adequate positioning of the marker ball in the X-ray as per standards above. The sources utilized for measuring these outcomes were Electronic Patient Record (EPR) and the Patient Archiving and Communication System (PACS). This data was then assessed on PACS system and was independently reassessed by a senior hip consultant to confirm whether it met the standard criteria. In order for a patient to have marker ball placement assessed adequately, both standards must be achieved.

The first audit cycle was completed between June 1, 2025, and June 30, 2025, where retrospective data was analysed from January 1, 2025, to May 31, 2025. Gaps in compliance against audit standards were identified and presented at a local clinical governance meeting, followed by agreement to aim for improving compliance. To achieve this, a visual reminder in the form of a poster (Appendix 1) was put up in relevant clinical areas, including the radiography trainees hub and X-ray station hub. Furthermore, a dedicated PowerPoint presentation (Appendix 2) by a Trauma and Orthopaedic Registrar was delivered to the Radiography team, focusing on the importance of including and correctly placing the marker ball during X-ray of the pelvis in an AP view. Moreover, a pioneer checklist was created, which was mandatory to be filled for all patients requiring the above-mentioned X-ray (Appendix 3). This included ticking off the standards performed, and a section for a valid reason for not being able to comply with the standards.

Data was collected, stored and analysed using Microsoft Excel (Redmond, WA, USA). Patient identifiers were removed from the datasheet prior to final analysis. Chi-square test was employed to statistically compare the results of the first and second loop result, with a p-value of less than 0.05 being set as a mark of significance. 

## Results

Sixty records met the inclusion criteria and were evaluated in the first audit cycle. Amongst these, only 45% (n=27) of patients physically had the marker ball present in the X-ray pelvis (AP view). Among these, 59% (n=16) of patients had the marker ball visible entirely and 41% (n=11) had the marker ball placed in the correct position. This yielded an overall compliance (both standards met in a single patient) of 59% (n=16). The results of the first audit cycle are given in Table 2. 

**Table 2:** First audit cycle (results)

Audit standard(s)	Observed compliance (%)	Targeted compliance (%)
Marker ball visible entirely	59% (n=16)	80%
Marker ball placed between both femurs and inferior to symphysis pubis	41% (n=11)	80%

Following the implementation of targeted interventions, a second audit cycle evaluating 50 cases was conducted. Compliance for the presence of marker balls was increased to 78% (n=39). The entire visibility of the marker ball increased to 84% (n=33), while the correct placement as per standards increased to 82% (n=32). This increased the overall compliance to 80% (n=31). The results of the second audit cycle are given in Table 3.

**Table 3:** Second audit cycle (results)

Audit standard(s)	Observed compliance (%)	Targeted compliance (%)
Marker ball visible entirely	84% (n=33)	80%
Marker ball placed between both femurs and inferior to symphysis pubis	82% (n=32)	80%

The second audit cycle showed improvement in both domains of the standards: an increase in compliance of the entire presence of the marker ball from 59% (n=16) to 84% (n=33) and the correct placement of the marker ball from 41% (n=11) to 82% (n=32). The educational interventions managed to achieve target compliance for both audit standards. 

Statistical analysis showed that improvement in standard 1 between the two audit cycles was statistically significant (p=0.042), while the change in standard 2 also showed a statistical significance (p=0.0014). The overall compliance increase showed a p value of 0.132. The results are presented in Table 4

**Table 4:** Statistical analysis * p value < 0.05 is considered statistically significant.

	First audit cycle (n=27)	Second audit cycle (n=39)	Chi-squared value	p-value*	Significant (Yes/No)
Marker ball visible entirely	16	33	4.12	0.042	Yes
Marker ball placed between both femurs and inferior to symphysis pubis	11	32	10.24	0.0014	Yes

Following the implementation of targeted interventions, including an educational presentation, poster prompts and a targeted checklist, the second audit cycle showed marked improvement.

## Discussion

This audit highlights the importance of quality control in ensuring correct marker ball placement, which can significantly impact preoperative templating prior to performing hip replacement in cases of intracapsular neck of femur fractures. The use of marker balls in digital templating for hip arthroplasty is well established as a method of accounting for radiographic magnification [10]. Several studies have highlighted that correct placement of the calibration marker is critical to ensure accurate templating. Boese et al. demonstrated that the marker ball position significantly influences digital templating outcomes, with misplacement leading to systematic errors in preoperative planning [10]. Similarly, Sinclair et al. found that in many cases, marker balls were either not visible or incorrectly positioned, which reduced the accuracy of templating for total hip arthroplasty [11]. 

The accuracy of templating also depends on the method of calibration. Bayne et al. evaluated the use of marker balls and showed that inaccurate placement could lead to clinically relevant errors, reinforcing the importance of standardised protocols [12]. Archibeck et al. further demonstrated that the use of marker balls provided greater accuracy than relying on assumed magnification, supporting their continued use despite practical challenges [13].

Newer approaches to calibration have been explored. Warschawski et al. compared the single-marker and double-marker methods, reporting improved accuracy with dual systems [14]. More recently, Maatough et al. compared the King Mark™ dual-marker system (Brainlab AG, Munich, Germany) with traditional single-marker methods, showing a clear benefit of dual-marker calibration in terms of reproducibility and precision [15].

Despite these advances, marker ball placement remains a persistent issue in clinical practice. Holliday et al. reviewed preoperative templating techniques and highlighted the variability in radiographic protocols, which may contribute to poor marker positioning [16]. Blake et al. proposed a practical approach to standardising calibration, but this has yet to be widely adopted in routine practice [17]. 

With the use of new Exeter stems [5], the need for correct placement of the marker ball is even more imperative. These new stems are heavily dependent on correct marker ball placement as it can severely affect the magnification factor during preoperative templating. This can further cause intraoperative complications such as inaccurate offset prediction, leading to multiple trials, further leading to intraoperative fracture risk, as well as postoperative complications such as leg length discrepancy, due to wrong implant size [18,19]. 

Overall, this audit reinforces that correct marker ball placement, to allow for accurate templating preoperatively should be embedded as routine practice. Regular audit cycles, coupled with targeted education sessions, ensure the standards are upheld, transparency is maximised and patient safety is not compromised. It is to be noted that although X-ray radiation exposure is involved, the use of a marker ball during X-ray imaging has been deemed safe [20]. 

At the end, the limitations of this study need to be addressed. Both audit cycles included 60 or fewer cases each, which may limit the statistical power of comparisons between the initial and post-intervention groups. As the audit was conducted within a single hospital's orthopaedic department, findings may not be representative of national or international standards. The lack of inter-observer reliability testing also renders a source of bias in the study design. The audit focused on process compliance rather than patient-centred outcomes, which would add to the value of ensuring proper marker ball placement for AP radiographs. While the educational intervention yielded short-term improvements in compliance, the long-term sustainability of these changes remains unclear, and therefore, further audit cycles down the line would help highlight this domain and are recommended.

## Conclusions

This audit demonstrated that simple yet cost-effective educational interventions can potentially improve the adequacy of marker ball placement in preoperative pelvic X-rays. This reflects greater awareness among radiographers and promotes safer clinical practice. Correct placement of marker balls are is only essential for accurate preoperative planning and management of intracapsular neck of femur fractures, but also provides critical protection in the medico-legal context.

Sustaining these improvements requires regular audit cycles, ongoing education and multidisciplinary engagement. By embedding rigorous preoperative assessment and record-keeping into everyday practice, trauma teams can enhance patient safety, reduce complications and nurture a more accountable and transparent surgical environment.

## Figures and Tables

**Figure 2:**
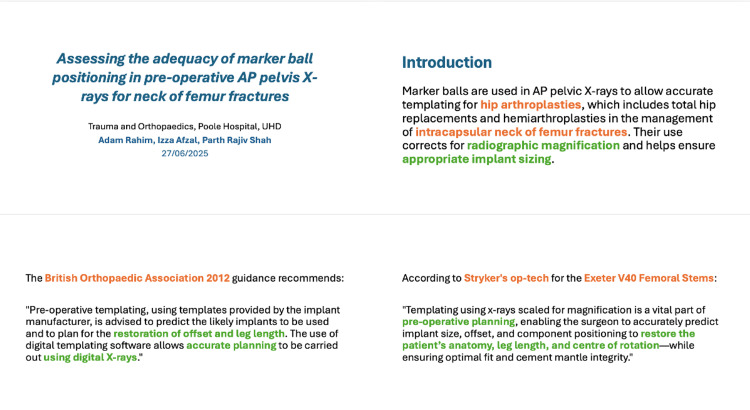
Appendix 2 (slides 1-4) Presentation disseminated to Radiology and Orthoapedic Team members, focusing on the audit results from first cycle, and increasing awareness on how to get an appropriately positioned marker ball in anteroposterior pelvic radiographs.

**Figure 3:**
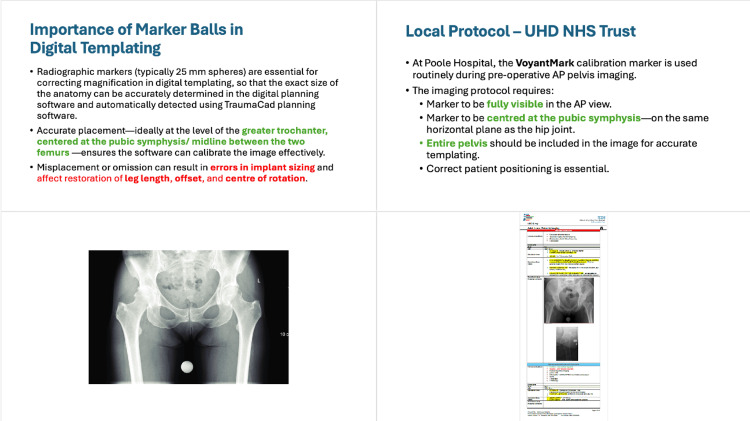
Appendix 2 (slides 5-8) Presentation disseminated to Radiology and Orthoapedic Team members, focusing on the audit results from first cycle, and increasing awareness on how to get an appropriately positioned marker ball in anteroposterior pelvic radiographs.

**Figure 4:**
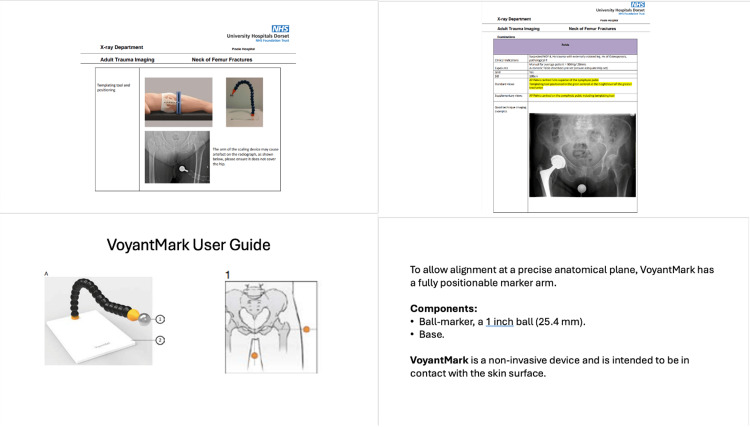
Appendix 2 (slides 9-12) Presentation disseminated to Radiology and Orthoapedic Team members, focusing on the audit results from first cycle, and increasing awareness on how to get an appropriately positioned marker ball in anteroposterior pelvic radiographs.

**Figure 5:**
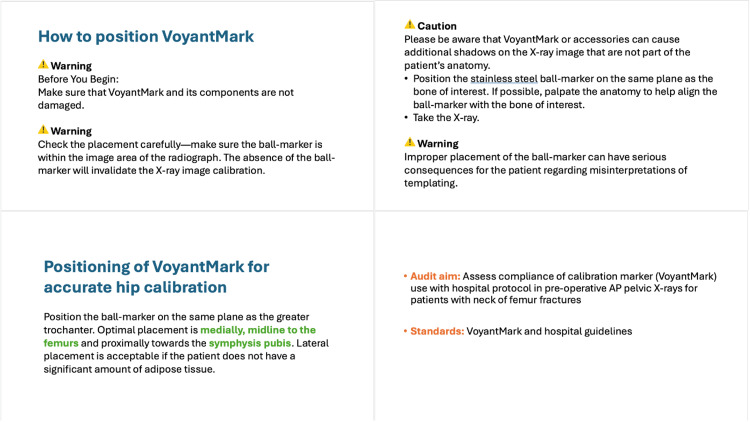
Appendix 2 (slides 13-16) Presentation disseminated to Radiology and Orthoapedic Team members, focusing on the audit results from first cycle, and increasing awareness on how to get an appropriately positioned marker ball in anteroposterior pelvic radiographs.

**Figure 6:**
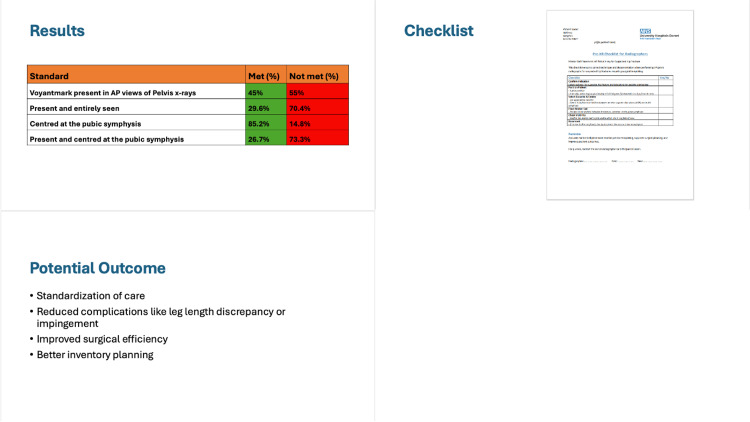
Appendix 2 (slides 17-19) Presentation disseminated to Radiology and Orthoapedic Team members, focusing on the audit results from first cycle, and increasing awareness on how to get an appropriately positioned marker ball in anteroposterior pelvic radiographs.

**Figure 7:**
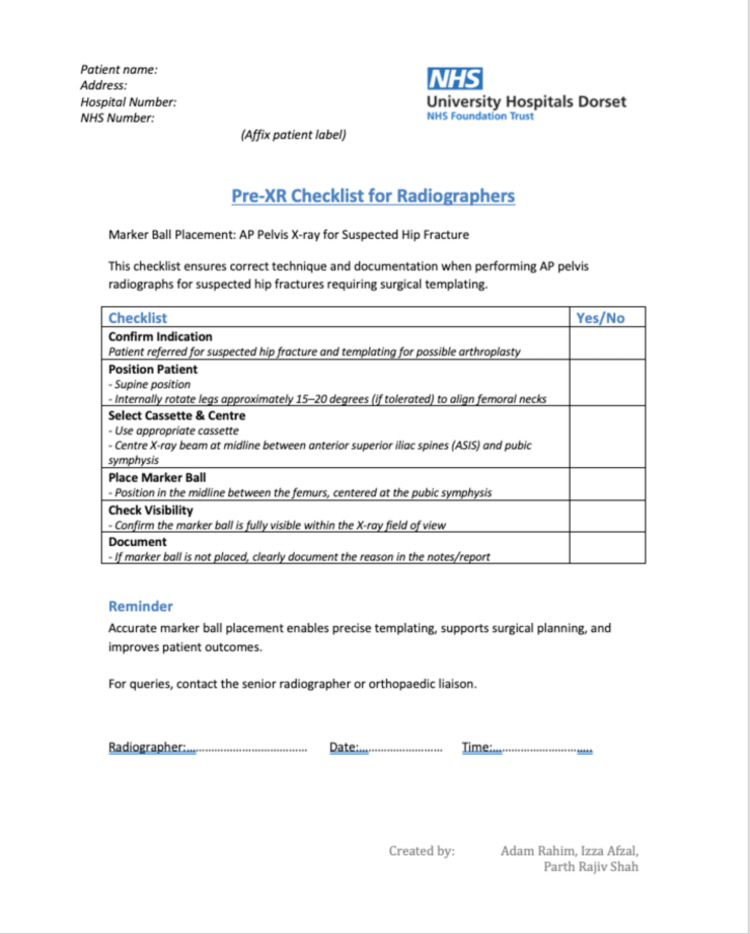
Appendix 3 (checklist) Checklist introduced for radiographers to ensure compliance with set standards for marker ball placement in anteroposterior views of pelvis.

## Appendices

Appendix 1

Appendix 2 

Appendix 3 
